# Supplementary figures and images for: A mathematical model for cancer risk and accumulation of mutations caused by replication errors and external factors
Source: PLoS One. 2023 Jun 14;18(6):e0286499. doi: 10.1371/journal.pone.0286499 (PMC10266611; doi:10.1371/journal.pone.0286499)

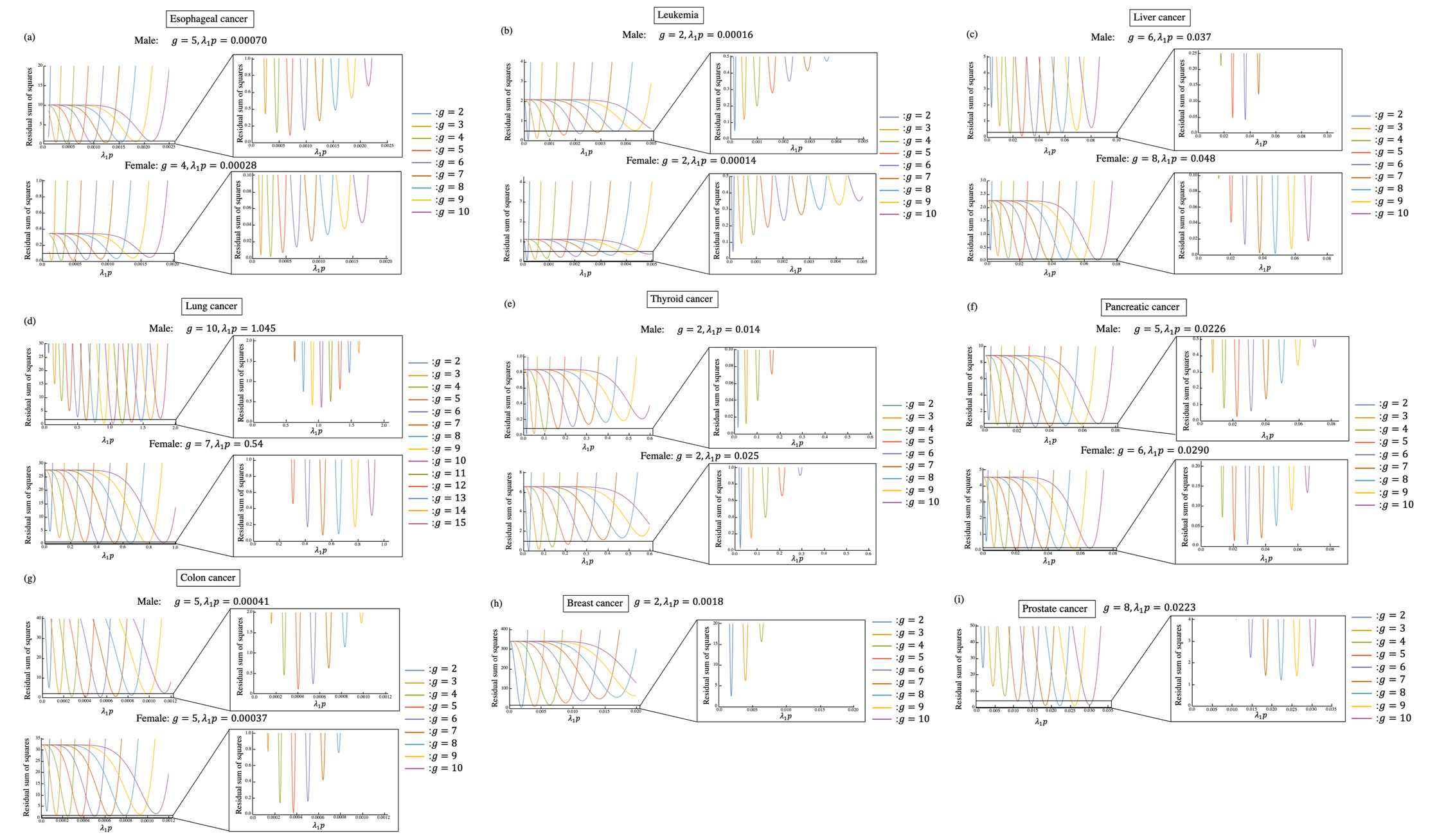

Supplement: S1 Fig — The sum of the squares of the residuals for each g was minimized by λ1p. Then, a combination of g and λ1p showing the minimum of the sum of squares of the residuals was established. The order of the figures corresponds to that in Fig 5: (a) esophageal cancer, (b) leukemia, (c) liver cancer, (d) lung cancer, (e) thyroid cancer, (f) pancreatic cancer, (g) colon cancer, (h) breast cancer, and (i) prostate cancer. The upper and lower panels show the results of males and females, respectively. (TIF) [file pone.0286499.s001.tif]
