# Supplementary material for: A mathematical model for cancer risk and accumulation of mutations caused by replication errors and external factors
Source: PLoS One. 2023 Jun 14;18(6):e0286499. doi: 10.1371/journal.pone.0286499 (PMC10266611; doi:10.1371/journal.pone.0286499)
Supplement: Attachment — Submitted filename: Response_to_Reviewers.docx [file pone.0286499.s003.docx]

>Reviewer #1: The described exercise investigates the multistage model, adding complexity in
>terms of including developing and stable tissue compartments. Overall the modeling lacks
>biological significance (i.e., no distinction regarding functions caused by mutations, assumes
>monoclonal tumor origin, assumes mutations rates that were not justified. Consequently, the
>value of the exercise is questionable. Also, identifying exogenously-induced mutations as
>wounds is non-standard nomenclature.

We appreciate the comments of this reviewer #1. We understand the concerns of this reviewer and revised the manuscript.

[Answers]
As reviewer #1 pointed out, this model ignores many biological processes. The goal of this paper is to examine how the cancer risks can be explained by biological processes as simple as possible. We couldn't express this point well, so we added descriptions (Line 82-85).

Although we ignored many biological processes, it is not difficult to incorporate some of the neglected processes. The model can include functions caused by mutations through parameters a_{i,j} and b_{i,j}(We added descriptions in Line 493-496, 500-502). However, it is very difficult to assume how mutations affect realistically. Therefore, for convenience, a_{i,j} and b_{i,j} were assumed to be constant. As the descriptions about this simplification were lacking, we added the explanation (Line 201-203).

As with the classic model such as Armitage & Doll (1954), this model also assumes a monoclonal tumor origin. This model is a monoclonal tumor origin because it assumes the population average in the carcinogenesis process. If we focus on individual cells, polyclonality can be expressed. We added a discussion about this (Line 496-500).

We added references and discussions on mutation rate (Line 449-459) and changed the term “wound” to “lesion”.

>Reviewer #2: Uchinomiya and Tomita present a mathematical model for accumulation of
>mutations during development and in adult tissues. They use it to fit cancer risk data, inferring
>that accumulation of DNA copying-errors is largely sufficient to explain cancer risk across
>many cancer types. Overall the study would appear sound, if the issues described below (1-3)
>are addressed. There is considerable interest in deriving these sorts of models for cancer risk
>and I think such studies could benefit from incorporating some cancer genomic data that is
>now available.

We appreciate the positive evaluation of our work by reviewer #2. We addressed all suggestions.

>Major:
>1- One major point of concern is that for the central result -- the fits to epidemiological cancer
>data (as shown in Figure 3) -- the inferred parameters "lambda_1*p" (number of oncogenic
>driver mutations acquired per cell division) and "g" (number of driver mutations required) for
>each cancer type were largely not constrained to match biological knowledge of each cancer
>type, nor were they checked to see if the inferred values match what we expect.
>There are some suspect examples where the "lambda_1*p" parameter can vary by >3 orders of
>magnitude between e.g. panel (g) [colon] and panel (d) [lung]. I think it is very unlikely tht the
>number of errors during cell division could vary so much between different tissues. The
>number of mutations per division has been measured recently by WGS for a variety of healthy
>human tissues; this data should be looked up and compared to the estimates of this
>parameter, to check veracity.

[Answer]
The mutation rate is estimated approximately 1.14 mutations per genome per division in healthy haematopoiesis and 1.37 per genome per division in brain development (Warner et al. 2020), which corresponds to lambda_1. Then, p can be interpreted as the proportion of cancer genes in the genome. Combined with the estimation that more than 1% of genes contribute to human cancer (Futreal et al. 2004) and the estimation that the protein-coding gene is 2% (IHGSC, 2004), lambda_1 * p is considered to be about 0.000228 or 0.000274. The values are too large in liver, lung, thyroid, pancreatic, breast, and colon cancer. This may suggest that biological processes other than replication errors affect the cancer risk. We added descriptions and discussions (Line 449-459).

>1b- A related point is the number of driver mutations in the "g" parameter: that this would be
>7-10 for lung cancer, 4-5 for esophagus, while 2 for thyroid and breast is a bit unusual. Does
>this match the actually observed number of drivers in these tissues in e.g. TCGA or similar
>cancer genomics data sets? (I appreciate it is not trivial to exactly measure the number of
>driver mutations per cancer type however some estimates can be made). If this doesn't match
>the models should be ajusted.

[Answer]
We referred to some papers for discussing the number of driver mutations. The parameter “g” of some cancers was not so different from the observation, but the others were different. Ignoring the biological processes in this paper may cause this mismatch, so we discussed it (Line 436-448).

>2- There is a worry about whether multiple solutions with different combinations of their 2
>parameters "lambda_1*p" (number of oncogenic driver mutations acquired per cell division)
>and "g" (number of driver mutations required) would fit the cancer incidence data similarly
>well, and thus might also be plausible solutions. Thus the fit might be visualized as a heatmap
>of the possible solutions. This fits the biological intuition that e.g. the somatic mutation rate
>will be somewhat different across individuals (they might have a germline defect in a DNA
>repair gene or not), and also that the number of driver mutations is variable within a cancer
>type -- it is a range, and not (as now somewhat artificially contstrained) a single value.

[Answer]
We agree with the concern that different combinations of the 2 parameters may be the solution. Although we tried to show it by heatmap, it were hard to see. Alternatively, we showed the relationship among residual sum of squares and the 2 parameters for each fitting (S Fig1).

>3- I think related with the above: there should be a formal test (perhaps based on bootstrap, or
>similar) to test if the addition of the lambda_2 parameter (mutagen exposure/wounds)
>significantly increases the fit of the models to the epidemiological data, or not. Now there is a
>statement about how the mutagen exposure factor is not necessary in the models to achieve a
>good fit to the cancer risk curves, however this doesn't seem to be supported with actual data.
>Does the fit improve with adding this (even if the improvement is subtle)?

[Answer]
The effect of the mutagen will be best understood in lung cancer, but since the fitting itself is successful even with the error, considering the effect of the mutagen may not improve the fitting. However, when considering only the duplication error, the estimated parameters are significantly different from the values known in experiments. When considering the mutagen, the parameters are close to the observed values to some extent. We added a figure and a discussion about this (Fig 6, Line 391-397, Line 482-491).

>Needs dissussing:
>- In at least some adult tissues, stem cells are not renewed during aging at the rate at which
>they are lost; their number declines with age. E.g. blood (HSC) is one example and there may
>be others. My intution is this should not impact their model massively. Can they
>comment/discuss, or reanalyze if necessary?

[Answer]
When the division rate decreases, the accumulation of replication errors in old age is overestimated in this model. It is necessary to consider the change in the division rate when constructing a more accurate model. We will be able to incorporate the effect by changing the division rate a_ {i, j} depending on time. We added a discussion about this (Line 502-507).

>- A tricky problem is that mutations resulting from DNA lesions ("wounds") might be occuring
>in a way coupled with DNA replication. Replication may increase the rate of converting wounds
>into mutations. If cells replicate slowly, there is plenty of time for repair e.g. via the NER
>pathway. If they replicate fast, lesions are not repaired but instead copied-over using TLS
>enyzmes thus generating mutations. So an ideal model would also account for this interaction
>between replication rate/ mutations and 'wound' exposures, having this as a parameter. I am
>not sure if such (more complicated) parametrization is feasible here, given the few amount of
>data points to fit to. Can they discuss this and/or adress with analysis if they think important?
>See the example of the "lambda_1*p" parameter (allegedly replication-dependant but not
>mutagen exposure dependant) being higher in lung; this is odd.

[Answer]
We are interested in the relationship between replication rate and lesions (we changed the term from “wounds”). The influence of radiation exposure on younger people is known to increase the cancer risk compared to adults. The relationship between replication rate and accumulation of lesions may also be important in discussing radiation effects. In terms of this model, it is conceivable to change lambda_2 with age or replication rate. We added discussions about this while addressing the comment above. (Line 503-507).

>- One idea for future work, which I understand might be out-of-scope here, is to model
>explicitly the existence of subsets of mutator cancers (e.g. MSI colon cancers; the BRCA breast
>cancer).

[Answer]
Indeed, this model only considers the average properties of cells in each tissue. In mutator cancers, lambda_1 related to the mutation rate will be larger in other cancers. I added a discussion about it (Line 459-467)

>Minor:
>- The mathematical formalism may be hard to follow for some readers without an appropriate
>background. Text describing the method is well described however takes effort to absorb. I
>suggest having a visual diagram/schematic describing the simulation method.

[Answer]
We added a schematic diagram as Fig1 and revised the numbers in other figures.

>- In figure 3, write cancer type on the figure panels. In Fig 2, write color legend in the figure.

[Answer]
We added a color legend in Fig 2(new number is Fig 3) and cancer type on the figure panels in Fig 3(new number is Fig 4).

>- Wording "Wound", while not incorrect, suggests a macroscopic event. In DNA, the word
>"lesion" is more commonly used.

[Answer]
We changed the term “wound” to “lesion”.

>- I don't understand much the emphasis on ionizing radiation in the abstract, while there are
>no analyses particular to this in the manuscript. It is also probably not a major carcinogen
>compared to e.g. tobacco smoking.

[Answer]
Our aim is to create a model for discussing the effects of low-dose and low-dose-rate radiation, which is difficult to observe. We emphasized that the model is effective for analyzing such phenomena. (L82-85)
