# Supplementary material for: A mathematical model for cancer risk and accumulation of mutations caused by replication errors and external factors
Source: PLoS One. 2023 Jun 14;18(6):e0286499. doi: 10.1371/journal.pone.0286499 (PMC10266611; doi:10.1371/journal.pone.0286499)
Supplement: Attachment — Submitted filename: Response_to_Reviewers.docx [file pone.0286499.s004.docx]

>Reviewer #2: The authors have adequately adressed my concerns with additional discussion.

[Answer] We were relieved that our response seemed to address your concerns. We have responded to all Reviewer #2’s comments.

>It is especially appreciated that they explicitly comment on the cases where their parameter fits are

>very different from realistic values, which may reveal interesting biology: "The estimated values of

>1 are too large in liver, lung, thyroid, pancreatic, breast, and colon cancer. This may suggest that

>biological processes other than replication errors affect the cancer risk." One final request I would

>have that this result " 1 are too large in liver, lung, thyroid, pancreatic, breast, and colon cancer",

>which is in my opinion important (not to invalidate the model but rather to suggest effects of

>additional biological factors), be shown in a separate figure or table. In other words, I would suggest

>to include a figure/table that compares the fitted parameters to a (range of) expected parameters

>from the literature, for each cancer type.

[Answer]We added the table comparing fitted parameters and expected parameters (Table 2).

>As a minor comment, the sentence in the abstract "The parameters estimated by the analysis of lung

>cancer data were closer to the observed values than when considering only replication errors." is

>quite unclear as written; consider expanding it to clearly convey what is the meaning/implication

>of this lung result.

[Answer]

We reconsidered the sentence (Line 33-34, 41-43).

>Finally it still unclear to me why the focus on radiation in the introduction if there is not any

>radiation specific analysis. It is not wrong, but is a bit confusing perhaps.

[Answer]

As we added in line 83-85, the carcinogenic effects of low-dose and low-dose-rate radiation have become a major public concern in Japan since the accident at the Fukushima Daiichi Nuclear Power Plant. The results of this paper are expected to make a significant contribution to the evaluation of cancer risk from low-dose and low-dose-rate radiation. Therefore, we would like to keep descriptions about radiation.

>Reviewer #3: The present reviewer has been added in the revision process. The study is well

>designed and addresses an interesting topic. However, there are several unclarities which need to

>be solved before acceptance for publication.

[Answer]

Thank you for your important comments. We have carefully considered and responded to those comments.

>Major comments

>1. Classically, as in ref. 2, the Armitage-Doll model is fitted to the rate (i.e., cases per person-year)

>unlike the present study, where the cumulative rate data (i.e., cases per total population) is used for

>fitting (equations 24 and 25). Please comment whether the “cumulative rate” data of the cancer

>registry herein are adjusted for competing factors (such as the age-related reduction in population).

>In addition, the cumulative rate at a certain age is dependent on the rates at all earlier time points

>because of its cumulative nature. This indicates that the uncertainty of the data is also accumulated

>with age. Fitting using the least square method assumes identical (i.e., age-independent) normal

>distribution on the uncertainty, and thus, does not take into account this cumulative nature of the

>uncertainty. Please consider stating these points, if applicable, as limitations of the study.

[Answer]

As Reviewer #3 pointed out, there are limitations related to demographics and the appropriateness of using the least-squares method. We added the discussion about these limitations (Line 519-528).

>2. In Fig. 2, the increase of N(t) in the growth phase seems to slow when it gets close to 300. This is

>not expected from the description of the model. If the authors modeled some slowing in the

>proliferation rate when the stem cell pool is nearly full, please comment on this in the Method

>section. If not, please explain the mechanism of the slowing.

[Answer]

Since the results are output when a certain number of divisions occurred for reducing the drawing cost, it looks that the rate of increase slowing around N=300 due to smoothing in combining them. We clarified in the caption that we are connecting discrete values. In addition, we did a new simulation and redrew the figure. In the new simulation, we set N=100 which used in figure 3.

>3. Equation 29 may need reconsideration. Because lambda_2 denotes the rate at which W changes

>in a cell, a differential equation of “dW/dt = lambda_2 * N_0 * exp[rt]” should hold. Solving this under

>an assumption of W(0) = 0, one obtains the equation of “W = (lambda_2 * N_0 / r) * (exp[rt] – 1)”

>instead of equation 29.

[Answer]

Thank you for your very helpful comments. We changed the approximation of W in the growth phase following the comments (eqs. 29 and 31). Following this change, the approximation of W in the stable phase must be changed, which was also addressed (eqs. 39 and 40). In addition, figures 3 and 5 are affected with this change and the Major-comment 4. We recalculated and redrew these figures.

>4. p.17 line 275 and equation 32. I do not understand why “a – b” is used here instead of “a”. Please

>consider using “a” or explaining why “a – b” is appropriate. I understand that the assumption

>described in lines 273-274 should be dealt with by the assumption of “a >> b” rather than the use of

>“a – b”.

[Answer]

We agree with this comment. Using “a” is more appropriate. We rewrote the related equations (eqs. 32-34, 36-38, 40). In addition, figures 3 and 5 are redrawn as mentioned in the response to the Major-comment 3

>5. In equation 40, the authors assume that exponential growth of the stem cell pool continues until

>age 18, which is quite counterintuitive, while this assumption contributes to the simplification of

>the model. Please discuss the consequence of changing the age at which the growth phase is

>terminated.

[Answer]

If the growth phase ends later, estimated g and lambda_1*p can be smaller. When the growth phase is long, the stable phase will be short, so it is necessary to develop cancer in a shorter time. Therefore, g should be small. Under this condition, cancer cells emerge earlier if lambda_1*p remains unchanged. To prevent this lambda_1*p should be smaller. I added a brief discussion about this (Line 526-527).

>6. p.20 line 326 “lambda_1 * p is regarded as a single parameter in Eq (25)” This statement may not

>be obvious to readers because “lambda_1 * p” is not explicit in Eq (25). Please consider showing the

>relevant calculation process.

[Answer]

We can show this by combining eqs (25) and (37), so we added the explanation (Line 343-345).

>7. Fig. 4 and S1 Fig. indicate different values of g between the sexes, which may seem odd. Please

>consider using an identical value for both sexes or showing a rationale for using different values

>(such as previous observations supporting a sex-dependent number of driver gene mutations).

[Answer]

We believe the value of g should be the same for both sexes. We think that there are two factors that cause the differences in the estimation. One is that the fitting is incomplete as pointed out in Major-comments 1. Esophageal cancers and pancreatic cancers with small differences in g may correspond to this case. Another cause will be the limitation of considering cumulative cancer risk based only on replication errors. Estimated g of Liver cancer and Lung cancer differ more between sexes. In Lung cancer, the influence of smoking is well known as risk factor, and the difference in g between sexes became smaller when the influence of mutagen was assumed. We added it to the Discussion for clarifying about this (Line 507-510, 522-523).

>8. p.29 lines 472-474. Ovarian germ cells may not be the only cell-of-origin of ovarian cancer

>because somatic cells of the ovary and the oviduct can also be its origin. Please reconsider the

>context.

[Answer]

We deleted the notation about ovarian cancer.

>9. Please consider code sharing as stated in <https://journals.plos.org/plosone/s/materials-software-and-code-sharing>.

[Answer]

We attached the code of Gillespie algorithm simulation.

Minor points

>p.16 line 245 and p.19 line 305: The value of N_L seems to be 300 instead of 100, as the maximum

>value of N in Fig 2 is 300.

[Answer]

We did new simulation with N=100 and redrew Fig.2 as response to Major-comment 3.

>p.20 line 314: The ministry name should be followed by the country name.

[Answer]

The notation of the reference was specified, so we wrote the order as it is.

[Answer]

We have corrected all the following concerns pointed out in the minor points.

>p.3 lines 31-32 and other places: Please consider adding the word “previous” when referring to previous observations.

>Example: … the estimated parameters did not always agree with “previously reported values”. (p.3 lines 31-32)

>p.47 line 47: “appearance” should be “mortality” according to ref. 2.

>p.6 lines 74-75: Consider the following modification: “… and ionizing radiation contributes to the carcinogenic

>process by adding a few mutations”

>p.6 line 91: … due “to” replication errors …

>p.7 line 106, p.24 line 387, p.31 line 503: Replace the first comma (,) with a semicolon (;) (e.g., …; however, …).

>p.12 lines 184-185: “…, in the growth phase, …” This phrase should be deleted.

>p.12 line 186: The subscript g should be substituted with s (Gamma_g to Gamma_s).

>p.13 line 207 and other occasions: Because “oncogenes” do not include “tumor suppressor genes”, the wording here

>should be “cancer driver genes” instead of “oncogenes”. The same applies to many occasions in the manuscript.

>p.14 line 217: “… of having more than g mutations …” should be “… of having g or more mutations …”

>p.14 line 221: “cancer risk” should be “cumulative cancer incidence”

>p.16 line 252: n should be d?

>p.17 line 273: the other -> another

>p.19 line 308 and other occasions: The wording of “statistical data” is odd and should be replaced with expressions

>like “epidemiology data”, “real-world data” or “cancer registry data”.

>p.20 line 328: “value” -> “values”

>p.24 line 385: “larger” -> “smaller”?

>p.29 line 480: “with on time” -> “with time”?

>p.30 lines 501-502: The grammar of “… should be depends on …” needs reconsideration.
