# Supplementary material for: A mathematical model for cancer risk and accumulation of mutations caused by replication errors and external factors
Source: PLoS One. 2023 Jun 14;18(6):e0286499. doi: 10.1371/journal.pone.0286499 (PMC10266611; doi:10.1371/journal.pone.0286499)
Supplement: Attachment — Submitted filename: Response_to_Reviewers.docx [file pone.0286499.s005.docx]

>Reviewer #2: The authors have adequately addressed referee remarks. The study is ready for publication
>in my opinion.

[Answer] We were relieved that our response seemed to address your concerns.

>Reviewer #3: The authors have adequately addressed my comments, with following minor
>points left. I apology that some of these points had been missed in the previous round of
>review.
>1. Line 39: re-estimate -> re-estimated
>2. Eq 1, 2, 6, 7, 12, 13, 15, 16 and 17: lambda_2 is used in some places instead of lambda_1.
>3. Eq 22: i is used instead of j.
>4. Line 269: “These means” -> “This means” or “These mean”
>5. Line 303: ... the number of lesion“s” ...

[Answer] Thank you very much for your careful checking. We have corrected all the points you pointed out.
